# Commentary: Roles of the Cannabinoid System in the Basal Ganglia in Parkinson's Disease

**DOI:** 10.3389/fncel.2022.897930

**Published:** 2022-05-09

**Authors:** Panteleimon Oikonomou, Wolfgang H. Jost

**Affiliations:** Center for Movement Disorders, Parkinson-Klinik Ortenau, Wolfach, Germany

**Keywords:** cannabinoids, CB1 receptors, CB2 receptors, Parkinson's disease (PD), basal ganglia, RCT - randomized controlled trial, commentary articles

## Introduction

This is a review article on the biological role of the cannabinoid system in the pathophysiology of Parkinson's disease (PD). The main components of the endogenous cannabinoid system (ECS) are the following: (1) the endogenous cannabinoids (eCBs), (2) the cannabinoid receptors, i.e., CB1 and CB2, transient receptor potential vanilloid-1 (TRPV-1), G protein receptor-55 (GPR55), and peroxisome proliferator-activated receptors (PPARs), and (3) various enzymes responsible for the metabolism of cannabinoids, i.e., fatty acid amide hydrolase (FAAH) monoacylglycerol lipase (MAGL). These components are presented regarding their distribution and possible function in the basal ganglia and their pathways (Wang et al., 2022).

## Commentary on the Main Statements Regarding Clinical Implications

All compounds of ECS were presented to be involved equally in the pathophysiology of the basal ganglia, with CB1 and CB2 responsible for the main examined roles.

The authors of the article support the notion that CB1 activation reduces excitotoxicity and promotes neural regeneration, suggesting a possible neuroprotective role in PD based on animal models and cell cultures studies (Wang et al., 2022), however, there are no clinical studies on patients with PD confirming that notion.

Regarding the effects of CB1 on neurotransmission, enhanced CB1 receptor transmission in the striatum and in the output ganglia of globus pallidus (GP) internus/substantia nigra pars reticulata may alleviate PD symptoms, whereas if CB1 is activated in GP externus, it exacerbates PD symptoms (Wang et al., 2022). This dual mechanism may explain why a CB1 antagonist (SR 141716) could not improve the severity of motor symptoms and levodopa-induced dyskinesia (LID) measured with UPDRS III and IV in an exploratory randomized, double-blind, placebo-controlled (RCT) study on 24 patients with PD (Mesnage et al., 2004).

Furthermore, the interaction between CB1 and dopamine receptors is justified presented as complex. The authors mentioned that CB1 receptor activation can antagonize the effect of dopamine receptor agonists on the one hand, yet may also act as a downstream effector of D2 receptors, on the other hand (Wang et al., 2022), which makes the expected clinical outcome unclear regarding the possible activation or inhibition of CB1 on PD symptoms, as well as on the response and side effects of dopamine agonists in patients with PD receiving cannabinoids as medication. This may also explain why both the CB1 agonist and antagonist may have been involved with LID. Indeed, the authors also referred that in the interaction between CB1 and D1, D2 receptors can modulate the LID (Wang et al., 2022), but the exact mechanism is still not understood, which is reflected in the fact that experimental and clinical trials reveal contradicting results (Sieradzan et al., 2001; Carroll et al., 2004; Chagas et al., 2014). Additionally, the relevance of the TRPV1 receptor as a new therapeutic target against LID is still not clearly understood and the experimental results, including the interaction with CB1, also remain controversial. Moreover, the existence of heterodimeric phenomena between CB1 and D2 receptors should also be taken into consideration during the interpretation of an experimental study in this field.

CB2 receptors are mainly distributed in brain glial cells and neural precursor cells, and less in nerve cells. Therefore, the neuroprotective effect could be promoted by an inflammatory modulation mechanism with fewer central nervous side effects. Studies with *in vivo* cell lines and PD animal models support this notion (Wang et al., 2022). Nevertheless, the application of selective CB2 agonists or antagonists in clinical studies on PD has not been conducted yet to our knowledge. The involvement of PPARs together with CB2 regarding neuroinflammation should be taken into consideration, designing a rational study in this field.

Still, basal research is needed to clarify the effect of the other cannabinoid receptors, e.g., TRPV-1 and GPR55 on neural cells, and the concept of antioxidant properties of different cannabinoids, which could be receptor-independent. Moreover, the authors supported the thesis that eCB-mediated restoration of cortical striatal synaptic plasticity through retrograde messenger function contributes to the improvement in motor symptoms in PD (Wang et al., 2022). Yet, to our knowledge, reliable data, apart from the PD animal model, to support this thesis are still missing.

On top of that, the authors mention that modulating enzymes related to the metabolism of cannabinoids is a novel therapeutic strategy, which could produce fewer side effects and have more applications. In fact, inhibitors of FAAH or MAGL showed an anti-depressive and anxiolytic effect in mouse models through modulation of corticosteroid levels and neuroprotective function in cell cultures through modulation of synaptic function (Wang et al., 2022). However, for the possible implication in humans, studies on safety profiles, possible effects on neuropsychiatric symptoms, and the progression of PD are still needed. In an RCT with 47 patients with PD, the use of nabilone, acting as a partial agonist on both CB1 and CB2 receptors in humans, mimicking AEA, could reduce sleep and anxiety (Peball et al., 2020).

Regarding phytocannabinoids (present in the cannabis plant, e.g., THC and cannabidiol), the authors provide evidence from *in vitro* cell culture studies about neuroprotective effects with various mechanisms, also independent of CB1 and CB2 receptors. However, clinical trials failed to prove these effects given the limitation of the small number of enrolled patients with PD and the short duration (Wang et al., 2022).

## Discussion

The long-accepted saying “Whoever heals is right” by clinicians, such us, need to be better understood by carefully comprehending relevant works from basic science. We aspire to make sure that as many patients as possible will actually and reasonably experience a benefit. Having that principle, we thank the authors of this review for offering a huge and comprehensive amount of knowledge about the role of ECS in PD, presenting possible targets for medical intervention. We do believe that we are in the preliminary stadium of having insights into ECS on the basal ganglia. However, smartly designed clinical trials are desperately needed to prove the concept as well as examine the safety and effectiveness of a modulation of the cannabinoid system for the benefit of patients with PD.

## Author Contributions

PO: writing—original draft and writing review and editing. WJ: review and supervision. All authors contributed to the article and approved the submitted version.

## Funding

This study was supported by Förderverein Ortenau Klinikum Wolfach e.V.

## Conflict of Interest

The authors declare that the research was conducted in the absence of any commercial or financial relationships that could be construed as a potential conflict of interest.

## Publisher's Note

All claims expressed in this article are solely those of the authors and do not necessarily represent those of their affiliated organizations, or those of the publisher, the editors and the reviewers. Any product that may be evaluated in this article, or claim that may be made by its manufacturer, is not guaranteed or endorsed by the publisher.
